# Local delivery of minocycline-loaded PLGA nanoparticles from gelatin-coated neural implants attenuates acute brain tissue responses in mice

**DOI:** 10.1186/s12951-020-0585-9

**Published:** 2020-02-05

**Authors:** Alexander Dontsios Holmkvist, Johan Agorelius, Matilde Forni, Ulf J. Nilsson, Cecilia Eriksson Linsmeier, Jens Schouenborg

**Affiliations:** 1 Neuronano Research Center, Department of Experimental Medical Science, Faculty of Medicine, Lund University, Medicon Village, Building 404 A2, Scheelevägen 2, 223 81 Lund, Sweden; 2 Centre for Analysis and Synthesis, Department of Chemistry, Lund University, Box 124, 221 00 Lund, Sweden; 3 NanoLund, Lund University, Professorsgatan 1, 223 63 Lund, Sweden

**Keywords:** Neural interface, Minocycline, Gelatin, PLGA, Nanoparticles, Drug-delivery-systems, Biocompatibility, Tissue responses, Immunohistochemistry, Brain

## Abstract

**Background:**

Neural interfaces often elicit inflammatory responses and neuronal loss in the surrounding tissue which adversely affect the function and longevity of the implanted device. Minocycline, an anti-inflammatory pharmaceutics with neuroprotective properties, may be used for reducing the acute brain tissue responses after implantation. However, conventional administration routes require high doses which can cause adverse systemic side effects. Therefore, the aim of this study was to develop and evaluate a new drug-delivery-system for local and sustained administration of minocycline in the brain.

**Methods:**

Stainless steel needles insulated with Parylene-C were dip-coated with non-crosslinked gelatin and minocycline-loaded PLGA nanoparticles (MC-NPs) were incorporated into the gelatin-coatings by an absorption method and subsequently trapped by drying the gelatin. Parylene-C insulated needles coated only with gelatin were used as controls. The expression of markers for activated microglia (CD68), all microglia (CX3CR1-GFP), reactive astrocytes (GFAP), neurons (NeuN) and all cell nuclei (DAPI) surrounding the implantation sites were quantified at 3 and 7 days after implantation in mice.

**Results:**

MC-NPs were successfully incorporated into gelatin-coatings of neural implants by an absorption method suitable for thermosensitive drug-loads. Immunohistochemical analysis of the in vivo brain tissue responses, showed that MC-NPs significantly attenuate the activation of microglial cells without effecting the overall population of microglial cells around the implantation sites. A delayed but significant reduction of the astrocytic response was also found in comparison to control implants. No effect on neurons or total cell count was found which may suggest that the MC-NPs are non-toxic to the central nervous system.

**Conclusions:**

A novel drug-nanoparticle-delivery-system was developed for neural interfaces and thermosensitive drug-loads. The local delivery of MC-NPs was shown to attenuate the acute brain tissue responses nearby an implant and therefore may be useful for improving biocompatibility of implanted neuro-electronic interfaces. The developed drug-delivery-system may potentially also be used for other pharmaceutics to provide highly localized and therefore more specific effects as compared to systemic administration.

## Background

In order to understand how neuronal networks in the brain process information in the awake freely moving animals and how such processing is changed by e.g. learning or diseases, long-term and stable recordings of multiple individual neurons are needed. In principle, this can be achieved by implanting electrodes, often referred to as “neural interfaces”, into the neuronal networks of interest. However, electrodes implanted in the brain elicit tissue responses comprising glial activation leading to the formation of a glial scar and loss of nearby neurons. Not only do these reactions and loss of neurons trigger a reorganization of the networks to be studied, they also adversely affect the quality of neuronal recordings. Considerable efforts have therefore been made to minimize these tissue responses [1]. We have previously shown that the mechanical properties, such as flexibility, size, anchoring and specific weight of the electrodes are of key importance [2–5]. However, it was only when combining ultra-flexible mechanical properties with a gelatin embedding that the ubiquitous loss of nearby neurons was, for the first time, abolished [4]. Moreover, we recently found that gelatin coating promotes healing of the blood–brain barrier [6]. Nevertheless, glial cell responses remain to some extent in the immediate vicinity of the electrodes. An alternative and complementary approach is to reduce glial responses by coating the electrodes with anti-inflammatory pharmaceutics.

A potentially useful pharmaceutic is minocycline, a broad-spectrum antibiotic, which also has anti-inflammatory, immunomodulatory and neuroprotective effects [7–11]. It is assumed to act by inhibiting microglial activation and their subsequent release of proinflammatory cytokines [8, 12, 13] which may be neurotoxic [14]. Even though minocycline is the most lipophilic antibiotic within the tetracyclines, it needs to be administrated repetitively at relatively high doses by conventional routes to have effects on the brain due to restricted passage over the blood–brain barrier [15, 16]. Moreover, minocycline has multiple common adverse side effects [17]. To improve the therapeutic efficacy and to obtain highly localized effects with less risk of side effects as compared to systemic administration, locally delivered drug loaded biodegradable nanoparticles is an attractive option [18]. Poly (d,l-lactic-*co*-glycolic acid) (PLGA) nanoparticles have been widely used for a range of biomedical applications, such as drug delivery [19], vaccination [20] and immunomodulation [21]. There is minimal systemic toxicity associated with using PLGA even in the CNS as the polymer undergoes hydrolysis into lactic acid and glycolic acid, which are eliminated from the body through common metabolic pathways [22]. On this basis, we have previously developed biodegradable minocycline-loaded PLGA nanoparticles that provide a sustained release in vitro of minocycline for several weeks [23] thus spanning the period of the most intense tissue inflammation after implantation [24, 25].

Gelatin, a protein-based hydrogel with zwitterionic properties, has proven to be a useful biomaterial for controlled release of several biologically active molecules [26]. However, to obtain a sustained drug release from a gelatin coating it is necessary to crosslink the gelatin [27] which often involves harsh processing conditions for sensitive molecules like minocycline [28]. Furthermore, gelatin sufficiently crosslinked to ensure sustained drug release creates a long-lasting barrier between the implanted electrode and surrounding tissue, which can be detrimental for the electrode function [1]. A conceivable approach would thus be to use a non-cross linked gelatin coating as a carrier for drug-loaded biodegradable nanoparticles. As gelatin is quickly dissolved and degraded after implantation, the nanoparticles are released into the adjacent tissue.

The aims of the present study were to first develop a method for embedding minocycline-loaded PLGA nanoparticles into non-crosslinked gelatin-coatings and then to clarify whether the addition of the minocycline-loaded PLGA nanoparticles can mitigate the acute brain tissue responses.

We report a novel method, suitable for neural interfaces, to incorporate minocycline-loaded nanoparticles in non-crosslinked gelatin coatings and a significant reduction in acute microglia activation and astrocytic response nearby the neural implants in mice.

## Materials and methods

### Minocycline-loaded nanoparticles (MC-NPs)

The following materials were used for preparing MC-NPs: PLGA Resomer RG503H (Sigma-Aldrich, Sweden), minocycline hydrochloride (Sigma-Aldrich, Sweden), didodecyldimethylammonium bromide (DMAB) (Sigma-Aldrich, Sweden), sodium bis (2-ethylhexyl) sulfosuccinate (AOT) (Sigma-Aldrich, Sweden). All other chemicals and solvents used were of analytical grade. Milli-Q ultra-pure water (Millipore, Sweden) was exclusively used for the preparation of all aqueous solutions.

MC-NPs were prepared by a single oil-in-water based preparation technique previously developed by us [23]. In brief, PLGA (20 mg) and a hydrophobic ion-pair complex of minocycline/Ca^2+^/AOT (3% drug-to-polymer ratio) was dissolved in an ethyl acetate/methanol mixture (9:1, 1 mL) at room temperature (RT). The organic phase was added drop-wise to an aqueous solution containing a stabilizer (DMAB, 0.10%, 10^−2^ M Tris–HCl buffer, pH 7.4, 1.2 mL) under magnetic stirring. The two-phase system was emulsified by sonication for 10 min using an iced ultra-sonic cleaning bath (VWR USC 300 D, 80W at 45 kHz). To this emulsion, Tris–HCl buffer (10^−2^ M, pH 7.4, 12 mL) was added under constant stirring, which resulted in nanoprecipitation. The suspension was then kept in an open beaker with magnetic stirring overnight to evaporate the organic solvent. The suspension was finally frozen in liquid nitrogen and lyophilized at − 55 °C and 0.050 mbar for 24 h (Freezone 4.5 model 77,510, Labconco, USA). Mannitol (200 mg) and Pluronic F-127 (1%, 10^−2^ M Tris–HCl buffer, pH 7.4, 1 mL) (Sigma-Aldrich, Sweden) [29, 30] were added as cryoprotectants before freeze-drying to prevent the nanoparticles from agglomerating. These procedures were identical to those described in previous work in our laboratory [23] and result in monodisperse particles 220 ± 6 nm in size, with a polydispersity index (pdi) of 0.07 ± 0.04, zeta potential of 55 ± 4 mV, drug content of 1.12 ± 0.01%, entrapment efficiency of 43 ± 1% and in vitro drug release characterized by an initial burst of 20% of the drug load during the first 24 h followed by a sustained release over 30 days.

### Fluorescently labelled nanoparticles (F-NPs)

Alexa Fluor 568 (AF568) Cadaverine (Thermo-Fisher, Sweden) was conjugated to PLGA through an amide bound between the carboxylic terminus of the PLGA and the amine group on the fluorescent dye. First, the carboxylic group of PLGA was activated through the formation of its *N*-succinimide ester. Dicyclohexylcarbodiimide (11 mg, 53 μmol) and *N*-hydroxysuccinimide (NHS) (7 mg, 53 μmol) were added to a solution of PLGA (500 mg, 27 μmol) in anhydrous dioxane (7.5 mL) and stirred for 4 h at RT. Precipitated dicyclohexylurea was removed by filtration (0.45 µm syringe nylon-membrane filter). The activated polymer (PLGA-NHS) was precipitated with diethyl ether (10 mL) and then purified by dissolving in dioxane and precipitating with diethyl ether three times and finally dried under reduced pressure. PLGA-NHS (10 mg, 0.54 μmol) was subsequently dissolved in dimethyl sulfoxide (DMSO, 1 mL), then triethylamine (2.1 mmol, diluted in DMSO) and AF568 Cadaverine (0.44 mL, 0.54 μmol in DMSO) were added. The solution was stirred for 7 h at RT and then the solution was poured into anhydrous diethyl ether to precipitate the fluorescent polymer (AF568-PLGA). The precipitated polymer was dissolved in dichloromethane and some drops of a saturated solution of HCl in diethyl ether was added to neutralize the excess of triethylamine. AF568-PLGA was finally precipitated with methanol and purified two more times by dissolution/precipitation using dichloromethane/methanol, respectively.

F-NPs were prepared according to the particle preparation method described above with the alteration of using AF568-PLGA mixed with PLGA in the ratio of 1:3 (w:w) and omitting the minocycline ion-pair complex. The hydrodynamic size, pdi and zeta potential of freshly prepared F-NPs (before adding cryoprotectants) were determined by dynamic light scattering (Zetasizer Nano-ZS, Malvern Panalytical Ltd., UK), taking the average of three measurements.

### Gelatin coating and nanoparticle absorption

Stainless steel needles (100 μm diameter, Austerlitz minutiens, Agnthos AB, Sweden) were cleaned in ethanol and insulated with Parylene-C (Galxyl C, Galentis S.r.l., Italy) (4 μm thick layer) using a compact bench top coating system (Labtop 3000, Para Tech Coating Inc., USA). A 30 wt% gelatin (porcine, type A, 300 Bloom, Sigma-Aldrich, Sweden) solution was prepared by adding the gelatin to cold HEPES-buffered artificial cerebrospinal fluid pH 7.3 (aCSF) [31] and allowing the mixture to swell for 5 min before heating at 60 °C for 1 h under magnetic stirring. The gelatin solution was then aliquoted into glass vials and stored at 4 °C overnight. Prior to dip-coating, the gelatin solution was reheated and held at 50 °C for 30 min to get rid of any air bubbles formed. The Parylene-C insulated needles were then dipped 5 mm into the warm gelatin solution at an immersion and retraction speed of 600 μm/s (dip coating unit model HO-TH-01, Holmarc Opto-Mechatronics Pvt. Ltd., India). The gelatin-coated needles were air-dried for five min at RT and then stored in a dark enclosure containing silica gel beads (less than 1% humidity, Type II, Sigma-Aldrich, Sweden).

**Fig. 1:**
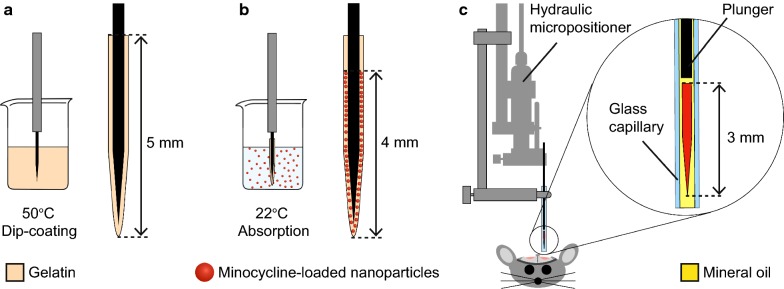
Schematic illustrations of dip-coating, nanoparticle absorption and implantation setup. **a** Dip-coating needle in gelatin (50 °C, down/up speed 600 μm/s); **b** absorption of minocycline-loaded nanoparticle suspension (RT, immersion time 30 s, down/up speed 600 μm/s); and **c** implantation setup with glass capillary and plunger mounted to a hydraulic micropositioner. Needle cut at 3 mm and glass capillary filled with mineral oil to avoid fluid uptake

Nanoparticles were embedded into the gelatin coating by an absorption method. One batch of freeze-dried nanoparticles (holding 20 mg PLGA and 0.6 mg minocycline) was first resuspended in 1.5 mL water by ultrasonication. Gelatin-coated needles were then immersed 4 mm into the nanoparticle suspension for 30 s at RT (immersion and retraction speed of 600 μm/s), allowing the gelatin to swell and absorb the nanoparticle suspension. The needles were air-dried for 5 min at RT and then stored in a dark dry enclosure (as described above). A schematic illustration of the dip-coating and absorption procedure is shown in Fig. 1.

### Coating characterization

The thickness of gelatin-coated needles before and after absorption of MC-NPs was measured at 3 mm distance from the tip using a DS-2MV digital camera (Nikon, Japan) mounted on a Nikon eclipse 80i microscope with a 10× objective. Swelling of the gelatin-coating was quantified by measuring the radial expansion over time after applying an aqueous solution containing cryoprotectants. Image capture and analysis were performed using the NIS-Elements 3.1 software (Nikon, Japan).

To visualize the absorbed nanoparticles, brightfield and fluorescent images of F-NP coated needles were acquired using the Nikon eclipse 80i microscope with a ×2 objective and NIS-Elements software. The radial distribution of F-NPs in the gelatin-coating was imaged using a Zeiss LSM 510 (Carl Zeiss Inc., Germany) scanning confocal microscope with an Acroplan 40×/0.8W objective. Two laser lines, 488 nm and 543 nm were used for sequential excitation of gelatin and F-NPs, respectively. Laser settings for each laser line was held constant for all images. Confocal images were collected using the Zeiss LSM 510 software (version 4.2, Carl Zeiss Inc., Germany). The intensity profile of a vertical cross-section at 50 μm depth at the 4 mm immersion boarder was quantified using open source software, FIJI [32].

The in-vitro release of F-NPs from coated needles was imaged using an Olympus IX51 microscope/DP21 digital camera setup (Olympus, Japan) with a 1.4 × objective. The coated needles were inserted (500 μm/s, using the same setup as for implantation described below) into an agarose-filled (0.2 wt % in aCSF) cuvette at RT. The radial spreading of F-NPs was measured using open source software, FIJI [32].

### Animals

23 transgenic mice (both male and female) that express green fluorescent protein (GFP) in brain microglia (B6129P-Cx3cr1, Jackson laboratories, USA) were used. All animals recovered from surgery with no apparent adverse effects and all survived for the duration of the experiment. The mice weighed 34 ± 6 g (mean ± standard deviation) at the time of surgery and they followed a normal weight curve post-surgery. All mice had free access to food and water and were kept in a 12-h light/dark cycle at a constant environmental temperature of 21 °C and 65% humidity.

### Surgery

Anesthesia was induced by placing the mice in a chamber of 2% isoflurane (Baxter Medical AB, Sweden) with 40% oxygen and 60% nitrous oxide. The head was shaved and subcutaneous injection of local analgesia (Xylocain, 3 mg/kg, Lidocain + Adrenaline, Dentsply Ltd., UK and Marcain, 1.5 mg/kg, bupivacaine, Aspen Nordic, Denmark), was given around skull and neck area. Also 0.5 mL of saline was given subcutaneously to prevent dehydration. The mouse was mounted in a stereotactic frame (KOPF Instruments, USA). A breathing mask (KOPF Instruments, USA) was attached for constant delivery of anesthesia. The concentration of isoflurane was gradually reduced to around 1% during the surgery. To keep the temperature stable a heating pad (3.7 × 14.5 cm, Homeothermic Monitoring System, Agnthos, Sweden) was used (body temperature was monitored using a flexible rectal thermal probe). After disinfection of the skin with 70% ethanol, the skull was exposed with a midline incision. The skin was retracted and the skull was cleaned from connective tissue under a stereomicroscope (M651, Leica Microsystems, Germany). Craniotomies (∅1 mm) were drilled midways between bregma and lamda, around 1 mm laterally of the midline, using a high-speed stereotaxic drill (Dest 300 IN, model MM 323IN, Silfradent, Italy). The underlying dura mater was left intact. The holes were rinsed with 0.9% sterile saline solution to clear away any possible debris and prevent the exposed tissue from drying. Care was taken to always keep a thin layer of saline on the cortical surface to prevent it from drying.

### Implantation

The needles were prepared for implantation by cutting them to a length of 3 mm and then placed inside a glass capillary (Microcaps, 1 μL, Drummond Scientific, USA). The glass capillary was mounted onto a hydraulic micropositioner (model 2650, KOPF Instruments, USA) together with a modified stainless-steel needle (250 μm diameter, Austerlitz, Agnthos AB, Sweden) used as plunger. The opening of the glass capillary was positioned over the craniotomy, approximately one millimeter above the brain surface. To avoid water uptake into the capillary during implantation and resulting swelling of the gelatin before entering cortex, the glass capillary was filled with paraffin oil (Sigma-Aldrich, Sweden). All needles were implanted, at a speed of 500 μm/s, to a depth of 3 mm below cortical surface. A schematic illustration of the implantation procedure is depicted in Fig. 1c.

After implantation, a drop of Agarose solution (2% in saline, type I-B, Sigma Aldrich, Sweden) was placed on top of the craniotomy and allowed to gel. The skull was then sealed using light-cured dental cement (RelyX Unicem, Elipar S10 (430–480 nm), 3M ESPE Inc., Sweden). The skin was closed over the dental cement using surgical clips. For post-surgery analgesia, Temgesic (0.1 mg/kg, buprenorfin, Indivior UK Ltd, UK) was given subcutaneously.

### Immunohistochemistry

At 3 or 7 days post implantation, the mice were deeply anaesthetized by an intraperitoneal injection of pentobarbital and transcardially perfused with 10 mL of saline, followed by 20 mL cold 4% paraformaldehyde (PFA) (Thermo Scientific Inc., USA) in 0.1 M phosphate buffer (pH 7.4). The brains were carefully removed by dissection and post-fixated in 4% PFA overnight (4 °C). For cryoprotection, the brains were further incubated in 25% sucrose until equilibrated. Subsequently, the needles were explanted, the brains were snap-frozen on dry-ice, horizontally sectioned (16 µm) on a cryostat (HM560, Microm, Germany), mounted (serially) onto glass slides (Super Frost plus, Menzel-Gläser, Germany) and stored at − 20 °C.

The sections were subsequently thawed and rehydrated in three 10-min washes of phosphate buffered saline (PBS). Thereafter, incubated in blocking solution, i.e. 5% normal goat or donkey serum and 0.25% Triton X-100 in PBS (1 h, RT), followed by incubation in primary antibodies overnight (blocking solution, RT). Primary antibodies included rabbit anti-CD68 (1:1500, Abcam Cat#AB125212) to identify activated microglia, rabbit anti-NeuN (1:500, Abcam Cat#AB104225) to identify neurons, and chicken anti-GFAP (1:1000, Millipore Cat#AB5541) to identify astrocytes. After overnight incubation, slides were washed (3 × 10 min in PBS) and incubated (2 h, RT) in blocking solution containing secondary antibodies. Secondary antibodies included donkey anti-rabbit Alexa Fluor (AF) 647 (1:500, Invitrogen Cat#A-31573), goat anti-rabbit AF594 (1:500, Invitrogen Cat#A-11037) and goat anti-chicken AF647 (1:500, Invitrogen Cat#A-21449). In addition, all tissue sections were stained with 4′,6-diamidino-2-phenylindole dilactate (DAPI) (1:1000, Molecular Probes Cat#D3571) to visualize cell nuclei. Finally, the slides were washed (3 × 10 min in PBS) and coverslips were mounted using polyvinyl alcohol mounting solution (PVA-DABCO, Sigma–Aldrich, Sweden). The slides were stored in 4 °C until imaging.

### Image acquisition and analysis

Fluorescent images of stained sections corresponding to a depth of approximately 400–500 µm into the cortex were acquired using a DS-Mv digital camera (Nikon, Japan) mounted on an BX53 microscope (Olympus, Japan) with a 20 × objective with identical lighting intensity and exposure time settings for each respective stain. Regions of interest (ROIs) were set to 0–50 μm and 50–100 μm from the border of the implantation site in each of the photographed sections.

To exclude unspecific staining in each individual image, intensity thresholds for CD68, CX3CR1-GFP and GFAP were set at 5.2, 7.3 and 3.8 times the background intensity, respectively. The immunoreactive response for CD68-, CX3CR1-GFP- and GFAP-positive cells was quantified by measuring the area fraction of the signal above the intensity threshold within each ROI.

The number of NeuN-positive cells (with a DAPI-positive nucleus as an inclusion criteria) was manually counted within each ROI and then expressed as number of neurons divided by the ROI area. Stained cells touching the ROI borders were assigned to the outer ROI on the right side of the image and to the inner ROI on the left side. DAPI-positive nuclei were manually counted in the same manner. Both image acquisition and analysis were done using the NIS-Elements 3.22 software (Nikon, Japan).

### Statistical analysis

Data from 31 successful implantations and subsequent histological procedures were used for statistics. From each implantation site and staining, the mean value of available tissue sections in the target depth was used in the statistical evaluation. Mann–Whitney test was used to compare the experimental groups within each ROI and time point, p-values < 0.05 were considered significant. All analyses were performed using the GraphPad Prism 8.0.1 software (GraphPad Software Inc., USA).

## Results

### Gelatin coating and nanoparticle absorption

Dip-coating the Parylene-C insulated stainless steel needles in gelatin resulted in uniform coatings with dry thicknesses of 4.8 ± 0.9 μm (mean ± standard deviation, n = 7). Characterization of the prepared minocycline-loaded nanoparticles (MC-NPs) including nanoparticle size (220 ± 6 nm), polydispersity index (pdi) (0.07 ± 0.04), zeta potential (55 ± 4 mV), drug content (1.12 ± 0.01%), entrapment efficiency (43 ± 1%) and in vitro drug release is described in previous work by us [23] (see also methods). To optimize the absorption of MC-NPs from the suspension, the swelling behavior of the gelatin-coatings was studied. The gelatin coating swelled 9.8 ± 0.4 (n = 3) times its original thickness after applying the aqueous solution and started to detach from the needle after 43 ± 3 s (n = 3). Therefore, the immersion time was set to 30 s. After absorption of the MC-NP suspension and drying of the gelatin coating, the coating thickness was 9.1 ± 1.2 μm (n = 7), that is almost doubled. The amounts of nanoparticles and drug absorbed in the coating can be estimated by considering the concentration of the nanoparticle suspension (20 mg PLGA and 0.6 mg minocycline in 1.5 mL water) and volume of the gelatin coating during absorption (85 nL). The coating was found to swell approximately 10 times its original size during a dipping cycle and, assuming unhindered diffusion of the suspension into the gelatin, a total amount of 1 μg nanoparticles and 34 ng minocycline is absorbed in the coating.

Fluorescently labelled nanoparticles (F-NPs) were used instead of MC-NPs for further coating characterization. Dynamic light scattering analysis of freshly prepared F-NPs (before adding cryoprotectants) showed a mean particle diameter of 150 ± 2 nm, pdi of 0.07 ± 0.01 and zeta potential of 62 ± 3 mV. These results are similar to the drug free nanoparticles prepared in our previous work [23], suggesting that the fluorescently labeled PLGA synthesized in this work did not interfere significantly with the formation of nanoparticles.

**Fig. 2:**
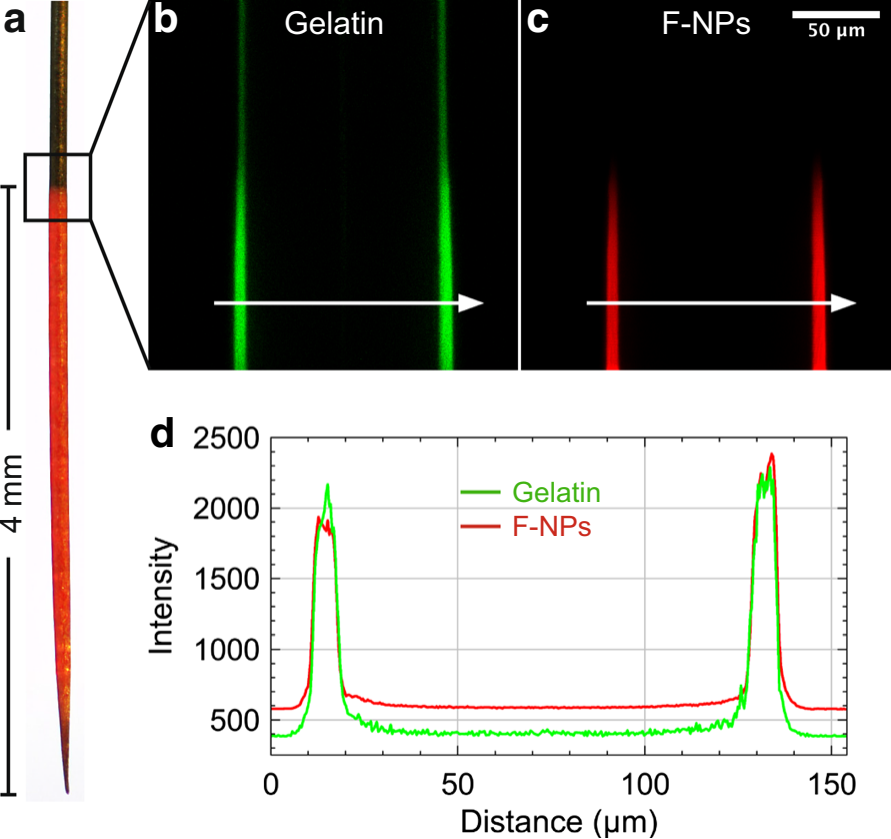
Coated implant with absorbed nanoparticles and intensity profile. **a** Brightfield and fluorescent image of a needle with F-NPs absorbed in the gelatin coating; **b**, **c** Two-channel confocal image (excitation wavelength for gelatin and F-NP was 488 nm and 543 nm, respectively) centered at the immersion border (box in **a**) and 50 μm depth; **d** fluorescent intensity profile of gelatin (green) and F-NPs (red) along the arrows in image (**b)** and (**c)**

The gelatin coating stayed intact during F-NP absorption and drying (Fig. 2a). The fluorescent intensity profile across the coating was found to be relatively even (Fig. 2b–d), confirming that the F-NPs were not only attached to the surface. Furthermore, no visible signs of agglomeration or clusters of nanoparticles were seen in the coating. The thickness of the coating was similar to that of MC-NP coating. This, together with similar particle properties (size, pdi and zeta potential), indicate that the drug-loaded nanoparticles were absorbed in the coatings to the same extent as the fluorescently labeled nanoparticles.

By insertion of the coated needles containing F-NPs in an agarose gel, it was confirmed that the gelatin coating stays intact during insertion. An additional movie file shows this in more detail (see Additional file 1). Moreover, as the gelatin started to swell, the F-NPs nanoparticles spread radially ∼ 850 μm from the implant over 10 min.

### In vivo effects on the brain tissue response

The tissue response to implanted needles coated with gelatin containing MC-NPs was evaluated at two different time points after implantation, 3 days (n = 9) and 7 days (n = 7), and compared to controls with gelatin-coated needles at 3 days (n = 7) and 7 days (n = 8). The expression of markers for activated microglia (CD68), all microglia (CX3CR1-GFP), reactive astrocytes (GFAP), neurons (NeuN) and all cell nuclei (DAPI) in an inner (0–50 μm from border of implant) and an outer (50–100 μm) ROI surrounding the implantation site was quantified. The diameters of the voids did not differ significantly between the groups (Kruskal–Wallis test).

#### Glial response

**Fig. 3:**
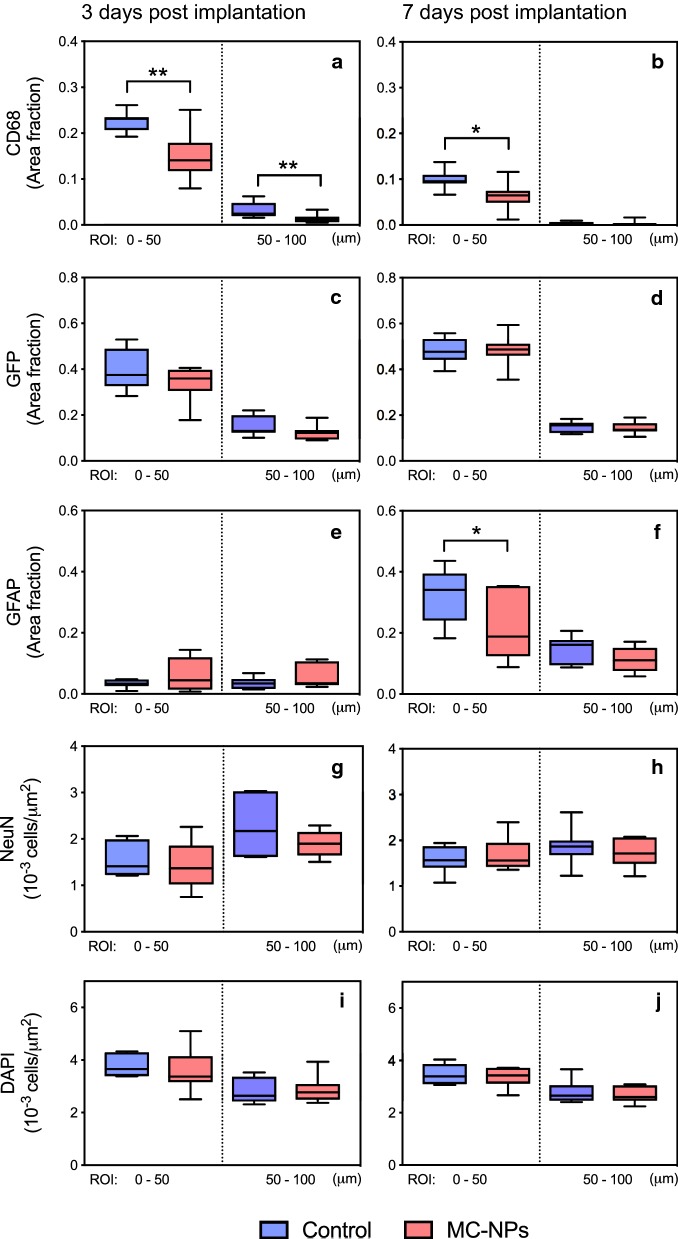
Quantitative analysis of the brain tissue responses at 3 and 7 days post implantation. Graphs with activated microglia (CD68) (**a**, **b**), CX3CR1-GFP positive microglia (GFP) (**c**, **d**) and the astrocytic response (GFAP) (**e**, **f**) are presented as the fluorescent area fraction whereas neuronal cells (NeuN) (**g**, **h**) and all cell nuclei (DAPI) (**i**, **j**) are presented as number of stained cells divided by ROI area. All graphs show the quantification of the inner (0–50 μm) and outer (50–100 μm) ROI. Boxes represent the first and third quartile with median values indicated as horizontal lines within each box and the whiskers show the minimum and maximum values. The horizontal brackets indicate statistically significant differences, p-values are labeled as **(< 0.01) or *(< 0.05)

**Fig. 4:**
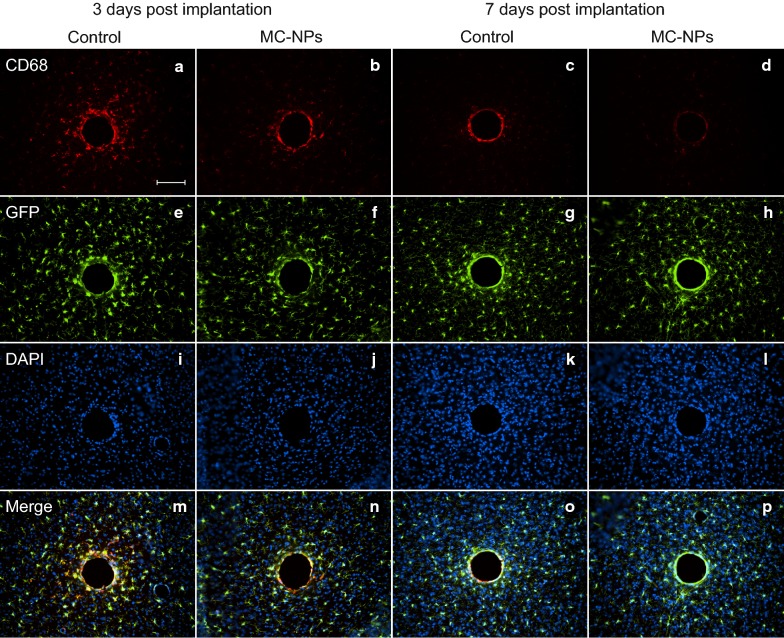
Representative immunofluorescent images of the microglial response. Tissue surrounding gelatin-coated needles with or without embedded MC-NPs at 3 days (two left columns) and 7 days (two right columns) post implantation. Images show activated microglia (CD68) (**a**–**d**), CX3CR1-GFP positive microglia (GFP) (**e**–**h**), cell nuclei (DAPI) (**i**–**l**), and merge (**m**–**p**). Scale bar = 100 μm

At 3 days post implantation, there was a significant reduction in CD68-positive cells around implants with MC-NPs compared to the control in both the inner (p = 0.0079) and outer (p = 0.0052) ROIs (Figs. 3a and 4a, b). After 7 days (Figs. 3b and 4c, d), the response had markedly decreased as compared to day 3 for both groups and had almost disappeared in the outer ROI. However, there was still a significant reduction of CD68-positive cells around implants with MC-NPs compared to the control implants in the inner ROI (p = 0.0289). As for the CX3CR1-GFP-positive cells (Figs. 3c, d and 4e–h), there was no significant difference between the groups at either time point. These observations suggest that MC-NPs selectively attenuates the activation of microglial cells without effecting the overall population of CX3CR1-GFP-positive cells around the implantation sites.

**Fig. 5:**
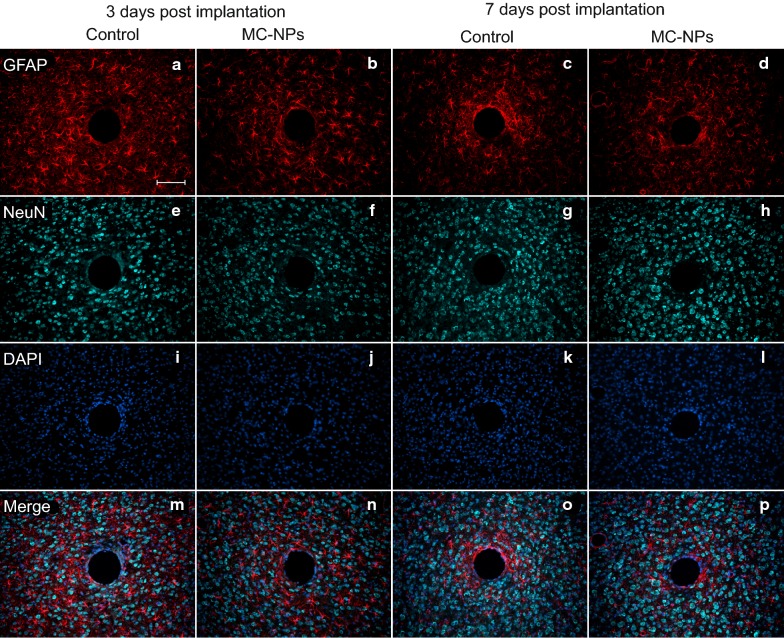
Representative immunofluorescent images of the astrocytic and neuronal response. Tissue surrounding gelatin-coated needles with or without embedded MC-NPs at 3 days (two left columns) and 7 days (two right columns) post implantation. Images show astrocytes (GFAP) (**a**–**d**), neurons (NeuN) (**e**–**h**), cell nuclei (DAPI) (**i**–**l**), and merge (**m**–**p**). Scale bar = 100 μm

The overall astrocytic response after 7 days (Figs. 3f and 5c, d) had increased for both groups in both ROIs compared to 3 days (Figs. 3e and 5a, b). However, there was a significantly lower astrocytic response around implants with MC-NPs compared to the control in the inner ROI (p = 0.0401) at 7 days, but not at 3 days.

#### Neurons and cell nuclei

The number of NeuN-positive neurons did not significantly differ between groups in either ROI or timepoints after implantation (Figs. 3g, h and 5e–h). The density of all cell nuclei (Figs. 3i, j and 4i–l) did not show any significant differences between groups within ROIs at either timepoint. These and above findings indicate that the nanoparticles do not influence neurons nor total number of cells up to 7 days in mice.

## Discussion

We have previously reported that gelatin can be used as a coating-material to provide structural support during implantation of ultrathin flexible electrodes in the brain [5, 33] and that gelatin itself can significantly reduce microglia activation but not the astrocytic response [4, 6]. Here we developed a novel method for local delivery of nanoparticles from gelatin coatings allowing the amount of drug to be kept several orders of magnitude lower as compared to systemic administration. Moreover, the locally delivered minocycline-loaded PLGA nanoparticles were found to considerably attenuate the acute activation of microglial cells as compared to gelatin alone and also cause a delayed reduction of the astrocytic response.

The early microglial activation at day 3 and subsequent astrocytic response on day 7 follows the normal inflammatory progression after insertion of devices into the brain [34, 35]. Microglia begin migrating towards the damaged site after about 12 h [36, 37] whereas astrocytes start migrating later. Upon microglia activation, pro-inflammatory cytokines are released which activate and recruit more microglial cells and appear to induce reactive astrocytes [14]. Conceivably, the substantial reduction of activated microglia (CD68-positive cells) by MC-NP in the present study is coupled to minocycline’s known inhibitory properties on microglia [8, 13]. There was no reduction of the overall microglial cells in the studied regions of interest, indicating no apparent effect of MC-NP on microglia migration. It remains to be determined, however, if the lowered astrocytic response at day 7 found in the present study is a direct effect from minocycline or a secondary effect from the decreased microglia activation. Reduction of both activated microglia and reactive astrocytes should be beneficial for neuronal survival as these reactive phenotypes are known to release components with neurotoxic effects [14, 38]. Nevertheless, we found no significant difference in the number of NeuN-positive cells between the MC-NP and control groups, suggesting that in this situation there is no significant neuroprotective action of MC-NP. This finding may be explained by the used study design with free floating implants and gelatin embedding, both of which known to be beneficial for neuronal survival [39, 40]. Hence, the need for neuroprotection is less. The lack of effect on neurons and overall microglia populations found here further suggest that the MC-NPs are non-toxic. However, there is a need for long term evaluation of the biocompatibility of MC-NP.

In the present study, we utilized the fact that gelatin swells but do not dissolve in water at room temperature. This made it possible to absorb the particles into the gelatin without compromising the thermosensitive minocycline and PLGA nanoparticles. Using non-crosslinked gelatin as the load-carrying coating material, has previously been shown to preserve the integrity of the neural implant without compromising its function [5]. In the present study, no traces of gelatin were seen in the surrounding tissue at either timepoints which is consistent with known rapid dissolution at body temperature and subsequent enzymatic breakdown into amino acids by upregulation of gelatinases (MMP-2 and MMP-9) in rodent brain [6]. The voids in the brain tissue remaining after explanting the implants were of the same dimensions as the implanted needles, which also suggest that the tissue contract tightly around the implant after the gelatin is dissolved.

It should be noted that the estimated amount of minocycline in each implant is less than 10^−6^ of the amount usually administered by conventional routes [9, 15], thus minimizing risks for unwanted side effects [17]. Furthermore, a sustained release from nanoparticles eliminates the need for repetitive drug administration [8].

The in vitro implantation video (see Additional file 1) also reveals that the gelatin coating creates a gelatin “track” in which the needle can be freely retracted, thus depositing a cylindrically shaped drug reservoir. This may be an interesting alternative to injection-based methods as it provides a well-defined local drug release.

## Conclusions

A novel drug-nanoparticle-delivery-system was developed for neural interfaces and thermosensitive drug-loads. The local delivery of MC-NPs was shown to attenuate the acute brain tissue responses nearby an implant and therefore may be useful for improving biocompatibility of implanted neuro-electronic interfaces. The developed drug-delivery-system may potentially also be used for other pharmaceutics to provide highly localized and therefore more specific effects as compared to systemic administration.

## Supplementary information

**Additional file 1.** In vitro implantation in agarose. Schematic illustration and video of the in vitro implantation of coated needles and spreading of fluorescently labeled nanoparticles in agarose gel.

## Data Availability

The datasets used and/or analyzed during the current study are available from the corresponding author on reasonable request.
